# A SWOT analysis of the development of health technology assessment in Iran

**DOI:** 10.1371/journal.pone.0283663

**Published:** 2023-03-30

**Authors:** Masoud Behzadifar, Mahboubeh Khaton Ghanbari, Samad Azari, Ahad Bakhtiari, Sara Rahimi, Seyed Jafar Ehsanzadeh, Naser Sharafkhani, Salman Moridi, Nicola Luigi Bragazzi

**Affiliations:** 1 Social Determinants of Health Research Center, Lorestan University of Medical Sciences, Khorramabad, Iran; 2 Health Management Research Institute, Iran University of Medical Sciences, Tehran, Iran; 3 Research Center for Emergency and Disaster Resilience, Red Crescent Society of the Islamic Republic of Iran, Tehran, Iran; 4 Hospital Management Research Center, Health Management Research Institute, Iran University of Medical Sciences, Tehran, Iran; 5 Health Equity Research Center (HERC), Tehran University of Medical Sciences, Tehran, Iran; 6 English Language Department, School of Health Management and Information Sciences, Iran University of Medical Sciences, Tehran, Iran; 7 Department of Health Education and Promotion, School of Health, Isfahan University of Medical Sciences, Isfahan, Iran; 8 Laboratory for Industrial and Applied Mathematics (LIAM), Department of Mathematics and Statistics, York University, Toronto, Ontario, Canada; Sichuan University, CHINA

## Abstract

**Background:**

Health systems need to prioritize their services, ensuring efficiency and equitable health provision allocation and access. Alongside, health technology assessment (HTA) seeks to systematically evaluate various aspects of health technologies to be used by policy- and decision-makers. In the present study, we aim to identify strengths, weaknesses, opportunities, and threats in developing an HTA in Iran.

**Method:**

This qualitative study was conducted using 45 semi-structured interviews from September 2020 to March 2021. Participants were selected from key individuals involved in health and other health-related sectors. Based on the objectives of the study, we used purposive sampling (snowball sampling) to select individuals. The range of length of the interviews was between 45 to 75 minutes. Four authors of the present study carefully reviewed the transcripts of interviews. Meanwhile, the data were coded on the four domains of strengths, weaknesses, opportunities, and threats (SWOT). Transcribed interviews were then entered into the software and analyzed. Data management was performed using MAXQDA software, and also analyzed using directed content analysis.

**Results:**

Participants identified eleven strengths for HTA in Iran, namely the establishment of an administrative unit for HTA within the Ministry of Health and Medical Education (MOHME); university-level courses and degrees for HTA; adapted approach of HTA models to the Iranian context; HTA is mentioned as a priority on the agenda in upstream documents and government strategic plans. On the other hand, sixteen weaknesses in developing HTA in Iran were identified: unavailability of a well-defined organizational position for using HTA graduates; HTA advantages and its basic concept are unfamiliar to many managers and decision-makers; weak inter-sectoral collaboration in HTA-related research and key stakeholders; and, failure to use HTA in primary health care. Also, participants identified opportunities for HTA development in Iran: support from the political side for reducing national health expenditures; commitment and planning to achieve universal health coverage (on behalf of the government and parliament); improved communication among all stakeholders engaged in the health system; decentralization and regionalization of decisions; and capacity building to use HTA in organizations outside the MOHME. High inflation and bad economic situation; poor transparency in decisions; lack of support from insurance companies; lack of sufficient data to conduct HTA research; rapid change of managers in the health system; and economic sanctions against Iran are threats to the developmental path of HTA in Iran.

**Conclusion:**

HTA can be properly developed in Iran if we use its strengths and opportunities, and address its weaknesses and threats.

## Introduction

Healthcare systems need to prioritize health services, ensuring efficient and equitable access and allocation of health provisions. This is fundamental in order to improve healthcare performance to promote and enhance health at the community/population level [1]. In recent decades, with globally changing political and social conditions, health systems have undergone extensive reforms to cope with complexities and new emerging situations and improve health, which represents a societal onus [2]. On the other hand, available resources for health systems are limited, and in order to provide appropriate health services, decisions must be made about the interventions required, and how to organize the activities of the health systems to meet societal needs [3]. Also, communities expect to receive high-quality services based on modern equipment in an affordable and sustainable way [4].

Health technology assessment (HTA) is a process that seeks to systematically evaluate various aspects of health technologies to be used by policy- and decision-makers in making appropriate, evidence-based, and effective decisions about a given technology [5]. HTA is intended to provide information on various policies and decisions in the health system and is a strong foundation in the field of health sector research [6]. Health policymakers are looking to devise and implement effective interventions, and, as a result, HTA has been increasingly used worldwide. In various health circumstances, HTA is a very effective tool for decision-making that helps prioritize interventions and resource allocation, and assess evidence using a consultative process with the cooperation of various relevant stakeholders [7]. HTA findings can help overcome uncertainties and facilitate decision-making in a transparent way based on evidence that is accurate, timely, and usable [8].

Different countries, based on their political commitment to achieve the goals of universal health coverage (UHC), need to decide on various interventions using currently available evidence [9]. HTA can be utilized as a tool to set priorities, and is an effective way to ensure achieving goals. Low- and middle-income countries (LMICs) are implementing various UHC-related programs as part of their efforts to achieve sustainable development goals (SDGs) by 2030 [10]. The lack of sustainable funding, human resources, and effective research are some of the challenges in moving toward UHC in these countries; also, the institutionalization of HTA has become a daunting task [11]. Therefore, the use of HTA in these countries has become increasingly important due to the efforts and need to properly inform UHC-related decision-making processes regarding the selection of health technologies and effective interventions based on available resources [12]. There is also a wide range of technologies and interventions in the healthcare sector that should be based on the optimal use of resources [13]. The use of HTA can improve the process of making appropriate decisions, as well as the design and implementation of health system policies [14, 15].

### The Iranian healthcare system

The public sector has the largest share in the delivery of health services and provisions in Iran [16]. The Ministry of Health and Medical Education (MOHME) is the main custodian of health and, in addition to training the human resources needed for health, is responsible for providing health services. Additionally, the government and insurance companies play a key role in financing the health system [17]. About 24,000 health centers across the country are responsible for providing primary healthcare (PHC) [18]. The PHC network is one of the most valuable achievements of the health system in Iran which has considerably improved the level of health in the country [19].

The MOHME is responsible for the identification of health needs, the implementation of health policies, and the delivery of healthcare provisions through an integrated network of medical, social, and educational services, aimed at reducing the burden of disease, and improving public and environmental health, as well as enhancing occupational health [20]. The MOHME oversees the activities of medical research institutions and units, monitoring research progress and outcomes, and validating and funding the programs of medical universities [21]. Moreover, the MOHME coordinates the activities of the PHC network, with a particular focus on maternal, child, and oral health. Finally, the MOHME is in charge of public assistance along with the development of the country’s health insurance system, which is one of the most important functions of any health system worldwide [22]. In Iran, both the public and private sectors are responsible for health insurance. Social security is the largest and most extensive insurance coverage program that provides insurance premiums through the participation of individuals. Military forces in Iran have also their own insurance program [23]. Finally, the Iranian Food and Drug Administration (FDA) is another governmental organization under the MOHME [20].

### HTA in Iran

Based on the decision of health policy-makers in Iran, HTA-related activities started in 2007 in the form of a Secretariat. The initial stages of its formation took place with the participation and support of researchers in order to receive requests concerning health technology evaluation and submission of reports [24]. From the beginning of 2010 and under the supervision of the deputy of treatments and the office of technology evaluation, standardization, and health tariffs, the HTA office resumed its activities with a new structure [25]. Health policymakers in Iran decided to train human resources with HTA skills, hence the Master’s degree program in HTA was established. Currently, four medical universities are responsible for educating students in the HTA field. Due to the role and position of policies related to drugs and other health products in Iran, the HTA and the economic management study group have been formed within the FDA [25].

We conducted the present study to identify the strengths, weaknesses, opportunities, and threats of the HTA program development and implementation in Iran. The results of this study can be a potential roadmap for health policy- and decision-makers in Iran.

## Methods

### Ethical approval

This qualitative study was conducted from September 2020 to March 2021. The study was approved by the ethical committee at Lorestan University of Medical Sciences with the following approval code: IR.LUMS.REC.1399.112. All methods were performed in accordance with the relevant guidelines and regulations. Also, we conducted this study in compliance with the Declaration of Helsinki. In addition, participants were informed of the purpose of the study, and were asked to consent to be audio recorded. All participants gave written informed consent before the start of the interview.

### Research framework

Strengths, weaknesses, opportunities, and threats were identified and analyzed using the SWOT analytical approach [26]. SWOT can help policy- and decision-makers in different sectors plan and implement various policies and programs as well as decision processes [27]. SWOT is a suitable tool for identifying internal (strengths and weaknesses) and external factors (opportunities and threats) that can affect the policy or program under study, and facilitate analysis of the subject and decisions [28]. The purpose of using SWOT is to show the positive forces that are involved in a policy or program, and the problems and challenges that need to be identified and implemented in the future [29]. This tool enables participants to share their views using a structured process. In this method, all features and relationships between internal and external factors of a phenomenon are considered [30].

In the existing scholarly literature, various studies related to HTA have been conducted using the SWOT framework. For instance, in a study conducted in Ukraine, SWOT was used to identify the strengths, weaknesses, opportunities, and threats of a proper creation and implementation of an HTA ecosystem in the country. The findings of this study showed that the establishment of a professional HTA organization based on the appraisal of the available body of evidence and the use of pharmacoeconomics is a strength, and the lack of a legal framework for establishing communication between HTA and stakeholders is a weakness. Creating a regulatory framework and developing plans for HTA communication with key actors was considered one of the most important opportunities [31].

In another study performed in Estonia, SWOT was used to assess a program aimed at developing HTA. The findings of this study showed that there are 16 strengths, 17 weaknesses, 17 opportunities, and 10 threats to establish HTA in this country. Some of the strengths included: providing access to information sources that others do not have and the multi-disciplinary nature of HTA. Mass media not being sufficiently involved and methodologies still evolving were deemed as weaknesses. Health reforms enabling the implementation of HTA-related activities and demand for transparency in decision-making were considered as opportunities, whilst depending on funding and unreasonably high expectations of HTA were seen as threats [32].

In Turkey, the SWOT framework was utilized to evaluate the development of an HTA program. The establishment of curated databases and information networks was considered a strength. Poor access to data or poor data quality, and traditional "expert-based" decision-making were identified as weaknesses. Increasing demand for the use of evidence and for transparency in decision-making was seen as an opportunity for the development of HTA in Turkey. The lack of dedicated funding was considered one of the most important threats in this study [30].

### Interview development

Based on the SWOT framework, a questionnaire including four questions was prepared. As a pilot, to meet the validity and reliability criteria, the texts of the interviews were sent out to five experts (see S1 Appendix). They approved the questions, which were used in the interviews.

### Participants

In-depth semi-structured interviews were used. Based on the objectives of the study, purposive sampling (snowball sampling) was used to select individuals. To maximize the data collected, as many participants as possible were interviewed.

Participants were selected from key individuals in the health and other health-related sectors. Individuals with 12 or more years of work experience and familiarity with HTA were eligible to participate. Some people did not participate in the study due to being too busy, not having time for interviews, and lack of interest. A total of 45 participants were interviewed. The overview of the participants is also presented in Table 1. Each participant was assigned a code. This study was conducted based on the location of the participants in different cities in Iran. The interviewers were selected to avoid any conflict of interest.

**Table 1:** List of participants enrolled.

Participants	No	Number of participants
Policy-makers	6	P1 to P6
Hospital managers	2	P7 to P8
University professors	5	P9 to P13
Iranian Red Crescent staff	1	P14
Health centre staff	3	P15 to P17
Researchers	4	P18 to P21
Faculty members	2	P22 to P23
Insurance managers	3	P24 to P26
Nurses	2	P27 to P28
Specialist doctors	6	P29 to P34
Pharmaceutical companies managers	4	P35 to P38
Graduate students in HTA	3	P39 to P41
Importers of medical equipment	4	P42 to P45

### Data collection

Before the start of the interview, an invitation email was sent by M.B. and A.B. to the participants, in which the objectives of the study and related upstream documents were contained. Later the questionnaire was sent. All the interviews were conducted and recorded by an experienced and educated team consisting of three people (M.B., A.B., and M.K.G.), who have Ph.D. in the health policy and health management fields and have several years of experience related to health sector issues, qualitative research and HTA. They are currently working in health-related research centers, and had no previous relationship with the participants.

M.B., A.B., and M.K.G. at the beginning of the interviews, explained the details of the interview and the reason for choosing the participant. Due to the COVID-19 pandemic, they had to have the interviews on Skype. All the interviews were conducted in a non-English language (Farsi) and, considering that the interviews were conducted through Skype, participants were interviewed based on their preference at their workplace or home. With the consent of the participants, interviews were recorded through field notes and participants’ voices. The interviews lasted between 45 and 75 minutes with an average duration of 55 minutes and continued until data saturation was reached. No repeat interview was carried out.

### Data analysis

After each interview, their text was transcribed word-for-word by hand and then typed. All interview transcripts were sent to the participants to confirm their accuracy and to ensure credibility and their feedback was collected. Regarding data saturation, a group discussion was conducted to achieve consistency among three researchers. As the original text of the interviews was in non-English language, their text was translated by a native author. If someone would like to have access to the original text of the interviews, they should contact the corresponding author.

For content analysis, Graneheim and Lundman’s strategy was used to analyze texts [33]. The steps of this strategy included: collecting the text of the interviews by the three researchers who conducted the interviews, writing the text of the interviews, initial coding of the texts, collecting the initial codes, and subdividing them according to the SWOT categories based on a careful comparative assessment in terms of similarities and differences. The data was, then, given to two researchers to review and agree on the method of coding. We finally prepared subsets based on the SWOT categories.

The transcripts of interviews were scrutinized by four authors (A.B., S.A, N.S, and S.M.) of the present study. Later, the data were coded according to the four domains of SWOT (strengths, weaknesses, opportunities, and threats). Data processing was performed using the MAXQDA software. In the next step, transcribed interviews were entered into the software. Also, all the steps including collecting, analyzing, coding, and placing based on the four SWOT items were reviewed by an external observer. Directed content analysis was also used for data analysis.

### Managing research bias

To improve the reliability of the results and reduce bias in data analysis, we conducted standard assessments such as data credibility, transferability, dependability, and confirmability. After collecting and analyzing the data, we sent out to all the authors via email a copy of the data analysis and classification of the interviews based on the SWOT framework. Also, we consulted with five experts to increase the validity of the findings and their interpretations. All the experts confirmed the findings. Meanwhile, to present and promote acceptable results in this study, a wide and diverse range of participants was considered.

The consolidated criteria for reporting qualitative research (COREQ) checklist were followed (see S2 Appendix) [34].

## Results

Table 2 shows the analysis of the interviews regarding HTA development in Iran, based on the four SWOT items. Participants mentioned 11 strengths for HTA in Iran: i) establishment of an administrative unit for HTA within the MOHME; ii) specific HTA-related university-level courses and degrees; iii) HTA being frequently used in decision making by some policy-makers and managers; iv) presenting HTA and its applications at national workshops and transferring knowledge; v) adapted approach of HTA models to the Iranian context; vi) HTA being mentioned and prioritized on the agenda in upstream documents, governmental bills and strategic plans related to the health sector; vii) scientific production and publications on HTA; viii) focusing on the efficiency of the health system among decision-makers and managers; ix) tendency to make optimal use of limited health resources; x) a gradual shift in the healthcare system toward evidence-based decision-making; and, xi) Health Transformation Plan (HTP) and other reforms in the health system (as overviewed in Table 3).

**Table 2:** Strengths, weaknesses, opportunities and threats of health technology assessment in Iran.

**Strengths**	**Weaknesses**
Establishment of an administrative unit for HTA within the MOHME University-level courses for HTA HTA is frequently used in decision-making by some policy-makers and managers. Presenting HTA and its applications at national workshops and transferring knowledge Adapted approach of HTA models to the context of Iran Mentioning HTA as a law on the agenda in upstream documents and government strategic plans (Health Sector) Releasing a specific scientific publication on HTA focusing on the efficiency of the health system among decision-makers and managers Making optimal use of limited health resources The movement in the health-care system toward evidence-based decision-making Health Transformation Plan (HTP) and other reforms in the health system	Unavailability of a well-defined organizational position for using HTA graduates HTA Advantages and its basic concept are unfamiliar to many managers and decision-makers Weak inter-sectoral collaboration in HTA-related research and Key stakeholders (in healthcare system (participation is weak or missed) Graduate students’ inadequate practical skills and shortage of HTA-trained staffs in MOHME, universities, and hospitals Underdeveloped electronic infrastructure in health system Processes for implementing HTA are not clearly defined and transparent Failing to consider all dimensions of HTA Inadequate engagement with international HTA organizations, and deployment of their knowledge and experience Not utilizing the media’s capability to introduce HTA Understanding the findings of HTA studies is not simple and clear or convenient for decision-makers. Lack of structural research related to HTA Insufficient participation of the private sector in HTA processes One-dimensional attention to HTA (policy-makers think HTA is only effective for awareness of clinical implications) Failure to use evidence-based decision-making in the health system Lack of adequate protection rules for HTA Failure to use health technology assessment in primary health care Weak capacity building for HTA
**Opportunities**	**Threats**
Support from the political side for reducing national health expenditures Commitment and planning to achieve UHC (on behalf of the government and parliament) Improving communication among all stakeholders engaged in the health system Decentralization of decisions and regionalization of decisions Increasing sensitivity of society and stakeholders for transparency in decisions and the use of HTA-based technologies and demand for new technologies Capacity building to use HTA in organizations outside the MOHME Increasing bargaining and reducing political lobbying Economic sanctions against Iran	High inflation and poor economic situation The desire to use luxury equipment without doing HTA Conflict of interest—poor transparency on conflicts of interest in the health system Poor transparency in EBM’s position in decisions Insurance companies do not support HTA research Increased demand for rapid introduction of technologies and their lack of evaluation Lack of sufficient data to conduct HTA research Changing the managers rapidly in the health system Lobbies in the health system—resistance of policy- and decision-makers to the evidence of HTA Some key managers do not believe in HTA and related recommendations Economic sanctions against Iran

**Table 3:** The subthemes and quotations of the strengths of developing the HTA in Iran.

Theme	Subtheme	Quotation
**Strengths**	Establishment of an administrative unit for HTA within the MOHME	Since there is an independent unit called HTA in the MOHME, it has caused managers and decision-makers to pay attention to this unit (P13). The structure formed for HTA is the proper step, but there is a need to strengthen its communication (P25).
	University-level courses for HTA	Training an HTA student can be very helpful in introducing it; also, a skilled workforce can increase HTA-related activities (P38). Now every province can benefit from the capacity of HTA graduates, and these graduates can adjust national policies according to the provincial context (P3).
	HTA is frequently used in decision-making by some policy-makers and managers.	In recent years, managers, decision-, and policy-makers in the health system have shown interest in using HTA and using its reports (P14). Some managers who passed HTA courses in other countries or had the experience in other countries highlighted the importance of HTA in the health system (P39).
	Presenting HTA and its applications at national workshops and transferring knowledge	Various workshops have been organized by the MOHME and the National Institute for Health Research that introduce the basics of HTA. The workshops made people interested in doing HTA studies (P1). When the upstream managers hear the importance of HTA in the form of cost, they want it to be used in the collection under their management (P5). Although managers encourage their experts to attend the workshops, they should also think about providing benefits for experts so that he/she will be motivated to put the new information into practice and won’t view HTA issues as an additional responsibility (P40).
	Adapted approach of HTA models to the Iran context	In connection with HTA studies, MOHME has begun to design a model based on Iranian indigenous conditions. Of course, this model needs to be completed (P15). Our country has characteristics on the cultural, religious, and social fronts that HTA in European nations might not take into account (P7).
	Mention HTA as a law on the agenda in upstream documents and government strategic plans. (Health Sector)	In Iran, there are 5-year plans for the promotion of various fields. One of the good things in the health sector was the emphasis on the use of HTA in government documents, and health-related organizations have been obliged to use it (P2).
	Releasing a specific scientific publication on HTA	Tehran University of Medical Sciences established the first specialized HTA journal to encourage and introduce HTA in Iran, and to develop research related to HTA. Many Iranian researchers can publish their articles in this journal, and this has made them work harder in this field (P41).
	Focusing on the efficiency of the health system among decision-makers and managers	In recent years, the MOHME is working to improve processes, and has pursued policies aimed at improving the efficiency of the health system. Also, managers have become interested in improving performance and appropriate use of resources in their decisions; in this regard, HTA can be a very good lever (P16).
	Tendency to make optimal use of limited health resources	Financial resources in the MOHME are very limited, and everyone has to help in various ways to use these resources properly. In doing so, many countries use HTA (P3).
	The movement in the health-care system toward evidence-based decision-making	Decisions of managers and policy-makers in the health sector are becoming more evidence-based. Also, the MOHME seriously recommends the universities of medical sciences to make decisions based on scientific evidences, local facilities, and conditions (P42). The possibility of being held accountable for bad decisions is now greater, so managers pay more attention to evidence-based decision making (P17).
	Health Transformation Plan (HTP) and other reforms in the health system	After the implementation of HTP in Iran, the health status in Iran changed. By prioritizing out-of-pocket payments and greater use of health services, the Iranian political government has virtually provided a way to use health-related activities such as HTA. Many activities were covered; people and managers became more sensitive to what is being conducted in the health sector (P43).

Sixteen weaknesses in the development of HTA in Iran were also identified. Despite the training of human resources, there are no suitable organizational positions available for them, and the skills of many of these people are still inadequate. Many policymakers are unfamiliar with the basis and objectives of HTA and do not make much use of evidence based on HTA. Cross-sectoral collaboration is still inappropriate for the development and implementation of HTA. Also, cooperation with international organizations is not very noticeable. HTA-related research is not very popular in the health system and only focuses on its clinical aspects. The subthemes and quotations of the weaknesses of developing the HTA in Iran are presented in Table 4.

**Table 4:** The subthemes and quotations of the weaknesses of developing the HTA in Iran.

Theme	Subtheme	Quotation
**Weaknesses**	Unavailability of a well defined organizational position for using HTA graduates	Annually, 12 students receive the master’s degree in HTA in Iran, many of whom cannot find a job. There is still no clear organizational position for those with a degree in HTA, and their skills are not used properly (P18). HTA graduates should present the outcomes of their studies and actions in a way that management will find more appealing (P44). Some managers do not even know that such graduates exist (P11).
	HTA Advantages and its basic concept are unfamiliar to many managers and decision-makers.	Despite the efforts of the MOHME to introduce HTA and its uses in the health sector, many managers and policy-makers are still unaware of its applications (P4). The managers of the prior generation, who typically own a significant amount of traditional power, often do not know HTA and its advantages (P19).
	Weak inter-sectoral collaboration in HTA-related research and Key Stakeholders (in healthcare system (participation is weak or missed	There are various health organizations in Iran, but there is no significant cooperation among them regarding HTA. Also, the MOHME is the only player in this field, and other organizations are not very aware of HTA (P45). The implementation of HTA necessitates that various stakeholders take on additional responsibilities and tasks, such data collection, etc., which they typically decline to do (P20). Various stakeholders and individuals must be involved in conducting the HTA research. In Iran, there is no real team in this field, and most of the actors in the MOHME do on their own (P9). In the Iranian health system, patients are not involved in the decision-making processes. All decisions are made without considering patients; this must be corrected (P21).
	Graduates’ inadequate practical skills and Shortage of HTA-trained staffs in MOHME, universities and hospitals	Many HTA graduates do not have sufficient skills to conduct HTA-related research. Although they have learned the basics, they are unable to do a systematic review, meta-analysis, or economic evaluation (P11). At the level of MOHME and universities of medical sciences, there is a shortage of experts (staffs) who are familiar with HTA processes, and many of these experts do not even know what HTA is (P6).
	Underdeveloped electronic infrastructure in health system	In recent decades, efforts have been made to build electronic infrastructure in the health sector, but not yet enough and this has led to problems in various activities in the health sector such as HTA (P13). The most significant issue with the currently planned electronic health information and prescriptions is that the data cannot be aggregated.
	Processes for implementing HTA are not clearly defined and are not transparent	Although HTA has been introduced and developed, there is still no process for its use in the MOHME (P22). I did not see any transparency about where HTA should be used in decision-making and programs (P15).
	Failing to consider the all dimensions HTA	In Iran, I have not yet seen individuals paying attention to the multidisciplinary nature of HTA. Only one or two dimensions are considered that are more systematic review and economic evaluation (P19).
	Inadequate engagement with international HTA organizations and deployment of their knowledge and experience	In the EMRO region, HTA structures are not very formed; on the other hand, MOHME of Iran has little connection with famous HTA organizations in the world (P17). The surrounding countries lack a lot of HTA development. Sanctions and economic conditions have limited the ability of managers to travel to advanced nations under HTA (P23).
	not utilizing the media’s capability to introduce HTA	Today, social media can be a great opportunity to introduce HTA which has no place in this network. We could have used scientific social networks to introduce researchers (P21).
	The Understanding the findings of HTA studies is not simple and clear or convenient for decision-makers.	For many researchers, HTA is a complex issue. No significant attempt has been made yet to introduce its findings. Simple HTA findings should be presented to decision-makers (P24).
	Lack of structural research related to HTA	HTA research is conducted in this area and most of the doses are in the field of medicine. Also, its various aspects have not been considered (P25).
	Insufficient participation of the private sector in HTA processes	In the health sector of Iran, the private sector has little place, and is mostly active in the treatment of patients in private hospitals. They have to work with the MOHME on HTA for many of the products that the private sector imports (P7). NGOs in Iran do not get involved in the proper use of equipment, drugs, and processes. The MOHME could use the capacity of NGOs, but is not very willing to take them seriously in the health system (P26).
	One-dimensional attention to HTA (policy-makers think HTA is only effective for awareness of clinical implications)	Since many people are unaware of HTA, they think they are only using HTA to see if it is a good treatment. Lots of things should be conducted in order to educate and raise awareness about HTA (P23).
	Failure to use evidence-based decision-making in the health system	Decisions in the Iranian health sector are not evidence-based, and are often personal decisions based on the managers’ own opinions. They must learn to use HTA according to the conditions of the health sector in Iran (P7).
	Lack of adequate protection rules for HTA	I have not seen any specific laws for the use of HTA in Iran, and the Parliament has not yet encouraged health-related organizations to use HTA as the main legislative body. There must be rules for using HTA (P31).
	Failure to use health technology assessment in primary health care	Although the PHC system in Iran is very extensive and has led to the improvement of indicators and health activities, HTA is not used at all in practice (P27).
	Weak capacity building for HTA	No attempt has been made by the MOHME to structure and streamline HTA in the body of medical universities, and administrators have made no effort to develop HTA in their decisions and activities (P28). There is no research funding for HTA studies in Iran, if any. Thus, researchers are reluctant to do research on HTA (P8).

The participants also identified opportunities for HTA development in Iran. The subthemes and quotations of the opportunities for developing the HTA in Iran are shown in Table 5. These included: support from the political side for reducing national health expenditures, commitment on behalf of the government and parliament and planning to achieve UHC; improved communication among all stakeholders engaged in the health system; decentralization and regionalization of decisions; increasing sensitivity of society and stakeholders for transparency in decisions and the use of HTA-based technologies and demand for new technologies; capacity building to use HTA in organizations outside of the MOHME; increased bargaining and reduced political lobbying; and economic sanctions against Iran.

**Table 5:** The subthemes and quotations of the opportunities of developing the HTA in Iran.

Theme	Subtheme	Quotation
**Opportunities**		
	Support from the political side for reducing national health expenditures	Political support for the health sector has increased, especially with sanctions that have reduced financial resources in Iran, and caused severe restrictions. The government has welcomed programs and activities such as HTA that can help optimize resource use (P29). As we hear in the media, the Parliament and the Judiciary are entering into how resources are spent in the health system, and some are even convicted (P33).
	Commitment and planning to achieve UHC (On behalf of the government and parliament)	Iran is also committed to UHC and, like many countries, wants to use its resources properly. In this regard, one of the HTA departments in the MOHME has used HTA research to increase programs in the three UHC areas (P39). If we look at the health plans that the leader of the country has outlined, we see links to HTA in articles 4, 5, 7, and 8 both directly and inferentially (P9).
	Improved communication among all stakeholders engaged in the health system	The presence of various actors and stakeholders to use resources in the health sector has increased in recent years. They are cooperating more; in addition to the laws that require them to work in the health sector, they try to organize their activities by interacting with other actors (P30)
	Decentralization of decisions and regionalization of decisions	By delegating many decisions to medical universities in the last two decades, decentralization has practically started. In this regard, managers must take into account their different environmental and local conditions in their decisions (P37).
	Increasing sensitivity of society and stakeholders for transparency in decisions and the use of HTA-based technologies and demand for new technologies	Like many countries, people and the health system are interested in using new technologies. Demand for the use of technology is increasing in Iran, especially in the health sector due to cultural and social issues. As a result, the presence of HTA activities is a very good opportunity to streamline these demands (P41). Iranians like to use the best and most up-to-date technologies in the health sector (P45).
	Capacity building to use HTA in organisation outside the MOHME	Through its activities, the MOHME is increasing the use of HTA for other organizations in the health sector. Many activities of the ministry such as importing medicine and equipment are subject to HTA (P31).
	Increase bargaining and reduce political lobbying	In recent years, especially in the field of equipment and medicines, the Parliament has enacted laws that medicines should be imported into country with no lobbies. Also, companies should follow the recommendations of the MOHME which is based on HTA (P10).
	Economic sanctions against Iran	At the same time, the scarcity of resources brought on by the sanctions has increased focus on strategies like HTA for the best use of resources (P2).

Finally, the subthemes and quotations of the threats to development of the HTA in Iran are presented in Table 6. These include high inflation and bad economic situation; the desire to use expensive equipment without doing HTA; conflicts of interest and poor transparency on conflicts of interest in the health system; poor transparency on decisions, which should leverage evidence-based medicine; lack of support from some sectors, including insurance companies; increased demand for rapid introduction of technologies and lack of evaluation and sufficient data to conduct HTA research; rapid change of managers and lobbying in the health system. Resistance and the disbelief of policy- and decision-makers to the body of evidence provided by HTA as well as economic sanctions were identified as threats.

**Table 6:** The subthemes and quotations of the threats of developing the HTA in Iran.

Theme	Subtheme	Quotation
**Threats**	High inflation and bad economic situation	In the last 5 years, there have been many economic problems in all sectors, which unfortunately have created many problems for the health sector. Also, the rising inflation has made difficult the use of facilities (P32).
	The desire to use luxury equipment without doing HTA	In Iran, there are many private companies that, regardless of economic and social conditions, import expensive equipment used in the health sector. They want to import the equipment of health sector and hospitals without considering HTA, and through some lobbies (P4).
	Conflict of interest -Poor transparency on conflicts of interest in the health system	One of the major challenges of the health system in Iran is the lack of mechanisms related to the conflict of interest. For example, many shareholders of private companies are also managers in the Iranian MOHME. They import a lot of equipment and medicines, and there is no transparent mechanism to investigate this issue (P6). HTA implementation involves a lot of conflicts of interest, and perhaps a lot of lobbying is done to prevent the implementation of even one study (P34). Conflict of interests is the missing link in decision-making in Iran. There are many managers who prefer their personal interests to organizational interests, and there are no practical’s rules for examining conflicts of interest in Iran (P8).
	Poor transparency in EBM’s position in decisions	It is true that in recent years the role of evidence has become more prominent. In the Iranian health sector, however, there is not much belief in the use of evidence in making decisions (P12).
	Insurance companies do not support HTA research.	The role of insurance in HTA processes is not significant. Despite many benefits that HTA can bring to insurers, insurance managers have a traditional look at health and insurance issues with no place for HTA (P35).
	Increased demand for rapid introduction of technologies and their lack of evaluation	Iranians are basically very much looking for luxury items. Therefore, companies import luxurious medical equipment, and physicians also use them. They insinuate that luxurious equipment and technology will help patients recover quickly (P10).
	Lack of sufficient data to conduct HTA research	The role of data in the Iranian health system is not significant due to the lack of sufficient attention to databases. Despite the fact that data plays an important role for every manager in making decisions, valid data have not been collected in the Iranian health system (P36). The data is in the hands of the insurance companies, and their managers believe that their management would come under scrutiny if the HTA inquiry reveals resource wastage (P14).
	Rapid change of managers in the health system	Managers in the health sector of Iran change frequently; thus, many things remain unfinished. When the new manager takes the position, he/she does not start from where the previous manager was (P12).
	Many lobbies in the health system- Resistance of policy- and decision-makers to the evidence of HTA	One of the major threats to the health system is the existence of power lobbies that, without considering the evidence and facts of the health system in Iran, impose high costs, and also cause facilities not to be used properly (P36). When researchers provide scientific evidence on a subject, in practice many managers resist it (P16). Even if you provide the appropriate HTA documentation to some managers, they may choose not to use it because they won’t be held accountable if they don’t (P20).
	Some key managers do not believe in HTA and related recomandations	The fact is that many managers do not pay attention to HTA at all. They do not want to consider research findings and evidence. It has been reported that 90% of managers act according to their interests, rather than scientific evidence (P37).
	Economic sanctions against Iran	Iran sanctions have affected the health system for many years, and have created many problems for health care providers and recipients in Iran (P18). The lack of resources brought by sanctions has also impacted the conduct of HTA studies. Managers prefer to utilize fewer innovations in these circumstances because the system stands less resilient (P40).

## Discussion

The existence of an HTA organizational unit in the MOHME has made it possible for policymakers to get acquainted with HTA activities. The MOHME attempted to plan a reliable, evidence-based decision-making system by establishing and making use of HTA [24]. Having units or organizations for HTA can enable the coordination of various activities, and facilitate their development and implementation [35]. Having such a structure determines how tasks can be categorized, segmented, coordinated, and which and how goals can be achieved in a better and faster fashion. In the Iranian health system, it was decided to structure its activities based on the findings and insights of HTA [6, 36]. Additionally, having an organizational structure for reporting and evaluating HTA is, indeed, crucial. In the absence of an HTA unit, only a committee of a few people would review the quality of HTA reports, and this would not provide opportunities for other groups and stakeholders to engage in in-depth reviews. This would also create serious problems and issues such as the lack of transparency. In a study conducted in 10 countries, the results showed that the presence of HTA agencies and units makes the processes of policies related to drug pricing and economic approaches more transparent, leaving less room to the application of administrative discretion in health policies [37].

It seems that in Iran, a larger institution needs to be established to provide clear and accurate evidence to decision-makers in addition to being in charge of HTA processes, including the opportunity to reconsider the technologies currently used in the health system as well as previous decisions, or provide evidence using HTA on technologies that have already entered the health system without HTA. One of the general policies related to HTA to increase awareness is to create different disciplines connected with HTA topics [38].

Studies from other countries show that low awareness of HTA and its benefits can lead to a limited understanding of HTA concepts and the evaluation of different interventions for the health system; also, their prioritization is not considered. For this reason, the lack of knowledge and basic understanding of HTA is one of the challenges of implementation of HTA in many countries [39]. Regarding HTA training, holding permanent workshops and intensive courses may play an important role. In many successful HTA departments, efforts have been made to increase officials’ skills through continuing education [40, 41].

Our study shows that one of the weaknesses of MOHME is the lack of awareness of its policy- and decision-makers about the basis of HTA and its valuable benefits. This is in line with other studies evaluating HTA in LMICs [30–32].

Although faced with the financial problems, the MOHME has attempted to use the mentoring capacities in the country to train skilled HTA-related manpower [6]. It seems that manpower training should be part of a structured HTA program [36]. This trend has been considered by policymakers in the Iranian health system, and so far, skilled manpower has started conducting HTA research. Also, multidisciplinary tools and methods must be developed to enhance evidence for decision-making, its use in evidence-based processes, and their transparency [42].

One of the problems in Iran is the lack of various skills among trained people. In Turkey, as in Iran, people are very well trained in HTA, but they do not have the necessary skills, and are not able to share their experiences [43]. Very good training in various fields of HTA is necessary, but it is not enough, and people should also apply their knowledge in a practical way so that their skills to perform HTA activities can increase. In order to solve the existing inefficiencies in Iran’s health system, the low skill level of trained people should be taken into consideration and HTA training should be dynamic. Moreover, this problem can be solved with face-to-face courses and regular conferences. Studies have shown that the lack of trained people and the existence of HTA low-skill professionals are the main obstacles to the use of HTA [44].

Using an HTA country-wide model locally run and informed by local, relevant data is fundamental in those countries that are heterogeneous in terms of wealth and size. In this regard, many countries draw on the experiences of developed and high-income countries in relation to HTA [45]. However, different contexts and settings that are country-specific should be considered for using HTA. For example, political, social, cultural differences, local, health, and economic data can affect it; therefore, HTA differences in different countries should be taken into account [8]. Paying attention to these differences can play an important role in the evaluation process of a given technology for decision-makers. In addition to using international evidence, it is recommended that local evidence should also be collected and considered in making decisions [46]. One of the activities related to HTA in Iran could be designing HTA processes based on the country’s conditions. Although these activities are not enough, they have been recently understood by several policy- and decision-makers.

In order to have HTA processes implemented in Iran, we should create a database of all the people who are trained in HTA and have different skill levels [47]. On the other hand, the establishment of an HTA center independent of the Ministry of Health can be effective in the development of HTA in Iran. The experience of some countries has shown that with the creation of such centers and assistance from international organizations in the form of meetings, conferences or symposiums, the development of HTA has accelerated. Collaboration between local and international researchers is effective for creating and supporting the institutionalization of HTA, and global partners can support the development of HTA and also raise its awareness in a country [48].

The use of evidence is a common practice in many health systems in recent years, and, in the Iranian health sector, interest in using evidence to make more appropriate decisions is growing. However, it is not yet pervasive in all areas; on the other hand, it seems that in the future decisions will be based on evidence. The use of HTA in Iran is expanding, and this is a promising event.

In many countries, policy-makers need to prioritize the use of health system budgets and try to make suitable investments in the health field. Also, they want these investments to have a real impact on health outcomes and improve health care delivery. In some countries, despite the policy-makers’ interest in using HTA, their willingness to use HTA is low [49]. In this regard, different actors and stakeholders should be included in the agenda, as it has been done in several countries [50].

The MOHME alone cannot provide all the skills that students need, and there must be cross- and multi-sectoral cooperation, especially with organizations that can improve students’ skills. Manpower also needs to increase skills to build credible evidence. Some countries, due to a lack of skilled manpower, have difficulties in building and implementing an HTA network, and, despite the availability of strong guidelines for using HTA, are unable to use it in their healthcare system [30, 51].

Different areas and subdisciplines/subfields can be considered in HTA, and this directly points to its multi-sector nature. The cultural, social, legal, ethical, economic, technical, and medical criteria of an intervention in HTA should be examined, requiring special skills and competences [52]. In the curriculum of medical and pharmacy programs, there are some courses on the clinical aspect of technology, but other HTA skills have not been considered [53]. Developing and poor countries should work to minimize the costs of inappropriate decisions in the health sector; in doing so, one of their problems is allocating sufficient economic-financial resources to HTA. Due to budget deficit, capacity building for HTA is faced with many problems.

Various medical, ethical, economic, and social implications must be considered in HTA. The views of other stakeholders and organizations should also be taken into account [46]. In addition, cross-sectoral cooperation should be considered for the development of evidence-based multicultural decision making, in which the views of almost all stakeholders have to be involved [54]. The MOHME in Iran is currently the most important actor in HTA, but alone it cannot provide all the context for HTA and other stakeholders must participate.

In connection with HTA, there are various stakeholders who can influence it depending on the political, social and economic situations. In Turkey, various policies have been implemented to attract the participation of different stakeholders, raising awareness, holding meetings and conferences to increase the orientation/attitudes of different stakeholders and their use of HTA for decision-making. There are stakeholders beyond the health sector who can make critical decisions that should be involved [30].

Meanwhile, there is no extensive cooperation to use all the potential of the financial capacity of the private sector in relation to health in Iran. The main activities of the private sector are related to equipment and medicines, and of course, HTA mechanisms have not been widely adopted by them [52]. The private sector has a strong desire to introduce various technologies into the health sector in Iran, and will also get many financial benefits, but the use of their financial resources for HTA research has been neglected. It can be an incentive to support and allocate funding for HTA if appropriate mechanisms are developed, requiring the private sector to submit HTA reports that are evaluated by policy-makers. Of course, all these mechanisms should be open and transparent so that they do not exploit HTA findings to their advantage [55].

The private sector is one of the most important users of HTA. Health policies and decisions play an important role in the adoption and use of new technologies. In a study, the findings showed that the private sector’s use of HTA in technology-related decisions was limited, and these results are consistent with our study. It is, indeed, necessary for the MOHME and other stakeholders to bridge the gap between the private sector and the government in order to increase the participation of the private sector [52].

To apply HTA, sufficient resources must be available. Without financial and human resources, as well as ongoing training programs, the quality of HTA processes might be affected [52]. Also, depending on the scope of HTA activities, researchers should be provided with appropriate funding. Various budget sources (public or private) should be allocated to HTA if the process of using HTA in a health system is to be supported by governmental regulations. Of course, with large private sector participation, a large portion of the HTA funding needs can be met. The use of HTA is a mandatory element for the use of new technologies, pricing, and refunding in the health system, and the private sector can, in whole or in part, accelerate this process [52].

In addition, HTA graduates do not have a good organizational position in the MOHME structure, and HTA is still unknown in most parts of it. Due to the lack of employment opportunities, graduates are forced to engage in activities that are not related to HTA, which means that their abilities have not been used [36]. Also, the lack of awareness has created many problems for the development of HTA in other countries [3, 56]. When a policy-maker has little knowledge of HTA, they cannot be expected to use it in their decisions. It seems that the development of HTA training is limited to the training of students, and still there is no suitable program for training managers.

Not only for HTA, but also in other parts of the health system, the lack of development of digital infrastructure and its use is a major weakness. Although some policies have been implemented to develop digital infrastructure, they are not enough, which has led to the lack of reliable and comprehensive data. In many cases, relevant data is missing or incomplete in connection with research activities that are part of the HTA ecosystem. The use of HTA requires adequate infrastructure and data to provide more logical and robust evidence [57, 58].

In some countries, data infrastructure is not suitable, and therefore many policy-makers use international data. This can affect decisions due to the differences between local and international data [58]. Information infrastructure and data facilities should be created in every country and serious attention should be paid to their development. Studies have shown that the existence of appropriate information infrastructure can help managers and policy-makers make clear decisions in using HTA [59]. Also, policy-makers need short and practical reports on how to use HTA findings [60]. There is still no consensus on the HTA reporting framework. Also, many policy- and decision-makers do not have much time to read long reports due to their busy schedules.

Political and organizational dimensions can also affect the use of health evidence. The allocation of resources and the availability of sufficient manpower cannot guarantee the use of HTA by policy-makers in their decisions; in this regard, a strong legal framework must be provided to build and strengthen capacity and demand for HTA [61]. This legal framework can also shape all HTA activities, and influence evidence-based decisions in all areas of health [62].

Widrig et al.’s study addressed the use of a given technology, and the analysis of legal issues that plays an important role in decision-making [61]. Findings showed that although legal issues are an important part of HTA, these issues are generally overlooked and should be included in HTA. Paying attention to the legal issues of HTA in Iran should also be taken into account; thus, preparing a framework can be useful in this regard [63].

Political support for the development of HTA can have important implications for evidence-based decision-making and resource allocation in health systems. Political support for HTA in Iran has been increasing, and the scope of decision-making based on strong, evidence-based, and verifiable criteria has clearly increased. Meanwhile, the use of HTA requires political willingness, mechanisms developed to use HTA, and appropriate legislation to reduce political resistance to HTA. HTA-related legal infrastructure is being established in Iran, and given its benefits, policy-makers are enacting appropriate laws to use HTA in various health sectors.

In 2014, the MOHME launched the HTP which was implemented with the aim of accelerating UHC. The implementation of this plan caused more policy-makers to pay attention to the health sector [18]. With the implementation of this plan, the role of evidence became more prominent paving the path toward using HTA.

Also, Turkey’s experience showed that with political changes and reforms in the health sector, the development and use of HTA increased [30, 64]. In one of the WHO reports, it is mentioned that there are HTA networks and institutions in the Asia Pacific region, and those few HTA agencies that operate in LMICs have problems due to lack of political support [65].

Given challenges such as Iran’s economic sanctions, declining revenues, and increasing health sector spending, policy-makers in various sectors, including the health sector, have implemented policies to manage resources, to get the most out of the health sector at the lowest possible cost [66]. In addition, decision-making in the Iranian health system is top-down; thus, the implementation of many policies is difficult due to the lack of attention to social, economic, cultural, and health differences in various provinces in Iran [67]. Therefore, in recent years, decision-making regarding some policies has been decentralized.

Changes in the government in Iran have provided the ground for HTA [17, 18]. Due to the lack of financial resources in the health sector, in the field of medicine and medical equipment, strict monitoring has been adopted for the introduction of new interventions. On the other hand, to expand their constituency, many members of Parliament lobby for various services and equipment to improve the health of the people in their constituency. In many cases, they receive equipment that is not necessary, and impose high costs on the MOHME. This is especially seen in connection with the import of various diagnostic devices. With the political changes in Iran, lobbying has also increased, and it seems that strong evidence must be provided for the use of various equipment, and the ground is also being prepared for the use of HTA.

While lobbyists cannot be prevented in Iran, policy-makers can be encouraged to use HTA in decision-making in order to use effectively health sector resources [68]. For example in France, with the development of HTA, the strong influence of political issues, and decision-making related to various interventions in health sector, the use of HTA has become a powerful lever in this country, strongly influencing decisions related to interventions that has led to the quality of healthcare provisions and public health [69].

Iran’s economic sanctions have caused high inflation and many economic problems for the people; also, the budget of many programs has been reduced [70]. Decreased funding means that decision-makers are not interested in using new technologies, and despite the existence of a new technology and HTA’s emphasis on using it, the health sector cannot have it.

On the other hand, the tendency to use expensive technology in Iran is expanding. Some individuals and companies import these technologies without considering HTA, which incurs high costs to the health sector and society. The greater danger is that they create a public demand for expensive and unnecessary technologies for the public sector that the public sector may not be able to use it [25].

Also, studies show that there is an increasing demand for the use of expensive and new technologies in the health sector, and policy-makers are not quick to approve them and limit their use [68]. There must be a road map to use new and expensive technologies, and HTA can be the best option. One of the major threats in all areas of health is the conflict of interest [68]. Especially in the field of medicine and equipment, this conflict causes several disadvantages. In Iran, too, many policy-makers have stakes in various health-related companies [71].

Also, strengthening HTA processes will make decisions become more transparent. Taking into account potential conflicts of interest in Iran’s health system has not received enough attention yet. In some cases, policy-makers have made decisions in their favor, and the uptake of technology innovations is directly related to their position or role. Various studies have shown that if the conflict of interest in decisions is reduced, HTA can become a more transparent and valuable option [68].

HTA is not just evaluation of technologies but can be used in various areas of health. The use of HTA in insurance and reimbursement issues is very important and it is applied in different countries [72]. However, in Iran, the use of HTA by insurers is not well accepted yet. Financial issues are one of the most important aspects of insurance. If insurers use HTA, they can also use its strengths in managing financial resources, reimbursement, and developing service packages [73].

Studies show that insurers have an important role to play in achieving UHC, and in this regard, they try to use HTA as a primary tool for preparing their lists of covered drugs and reimbursements [1, 74].

Although HTA and its role have been emphasized by decision-makers in various countries, there are still high-level executives who do not believe much in evidence-based approaches for the implementation of a program. In Iran, at different levels of managers, there are different views on the use of technologies and HTA [24]. Some policy- and decision-makers are against HTA and do not believe in it, potentially because of inadequate knowledge about HTA, or conflicts of interest [75].

Iran’s economic-financial resources for buying and utilizing expensive technologies are limited. Despite the achievements in improving the level of health, Iran still has a long way to reach a desirable state of health; therefore, Iran needs to use new technologies that help shortcut this path. The pricing logic of new technologies in Iran faces many challenges, and accordingly, health sector managers must make great efforts to obtain political support to enact HTA-related laws.

This study suffers from some limitations. We did not have access to all the experts in the field of HTA, so we could not get their opinions. Due to COVID-19, it was not possible to interview face-to-face and interact more appropriately with the participants. Another limitation of this study is that different points of view can be applied regarding the classification of items in the four SWOT categories, and an opportunity can be considered a strength by another person. Also, one person can see an item as a threat and another person as an opportunity. In this study, efforts were made to consider the most common opinions in such a way to reach a consensus.

## Conclusion

HTA plays an important role in the healthcare sector of all countries; also, health systems have been increasingly using it to manage their resources. Findings of this study show that, based on the SWOT analysis, in the development of HTA in Iran, four dimensions of SWOT should be considered. Findings also show that Iran has a long way to take for the development of a cutting-edge HTA. A wide range of strengths, weaknesses, opportunities, and threats were identified for a proper development of HTA that should be addressed by policy- and decision-makers.

## Supporting information

S1 Appendix The four questions of the questionnaire based on the SWOT framework, which was used during the interview. (DOCX)

S2 Appendix Consolidated criteria for reporting qualitative studies (COREQ): 32-item checklist. (DOCX)

## Abbreviations

SWOT: Strengths, weaknesses, opportunities, threats
HTA: Health technology assessment
COREQ: Consolidated criteria for reporting qualitative research
UHC: Universal health coverage
LMIC: Low- and middle-income countries
SDGs: Sustainable development goals
MOHME: Ministry of health and medical education
PHC: Primary health care
FDA: Food and drug administration
