# Supplementary material for: A SWOT analysis of the development of health technology assessment in Iran
Source: PLoS One. 2023 Mar 30;18(3):e0283663. doi: 10.1371/journal.pone.0283663 (PMC10062657; doi:10.1371/journal.pone.0283663)
Supplement: S1 Appendix — (DOCX) [file pone.0283663.s001.docx]

**Appendix 1:**

1. Describe the strengths of implementing health technology assessment in the Iranian health system.
2. Describe the weaknesses in the implementation of health technology evaluation in the Iranian health system.
3. What opportunities are there to implement health technology assessment in the Iranian health system?
4. What are the threats to implementing health technology assessment in the Iranian health system?
