# Supplementary material for: A SWOT analysis of the development of health technology assessment in Iran
Source: PLoS One. 2023 Mar 30;18(3):e0283663. doi: 10.1371/journal.pone.0283663 (PMC10062657; doi:10.1371/journal.pone.0283663)
Supplement: S2 Appendix — (DOCX) [file pone.0283663.s002.docx]

**Appendix-2:**

Consolidated criteria for reporting qualitative studies (COREQ): 32-item checklist

**Title:**

A SWOT analysis of the development of health technology assessment in Iran

Adapted from: Tong A, Sainsbury P, Craig J. Consolidated criteria for reporting qualitative research (COREQ): a 32-item checklist for interviews and focus groups. Int J Qual Health Care. 2007; 19(6):349-57.

| **No. Item** | **Guide questions/description** | **Reported on Page #** |
| --- | --- | --- |
| **Domain 1: Research team and reﬂexivity** |  |  |
| *Personal Characteristics* | | |
| 1. Inter viewer/facilitator | Which author/s conducted the interview or focus group? | Page 7  All the interviews were conducted by (M.B., M.K.G., and A.B.) |
| 2. Credentials | What were the researcher’s credentials? E.g. PhD, MD | Page 7  Three people, who have Ph.D. in the health policy and health management. |
| 3. Occupation | What was their occupation at the time of the study? | Page 7  They are currently working in health-related research centers. |
| 4. Gender | Was the researcher male or female? | Page 7  M.B. and A.B. are male and M.K.G. is female. |
| 5. Experience and training | What experience or training did the researcher have? | Page 7  The three researcher(M.B., M.K.G., and A.B.), have many years of experience related to health sector issues, qualitative research and HTA. |
| *Relationship with participants* | | |
| 6. Relationship established | Was a relationship established prior to study commencement? | Page 7  They had no previous relationship with the participants. |
| 7. Participant knowledge of the interviewer | What did the participants know about the researcher? e.g. personal goals, reasons for doing the research | Page 7  First, before the start of the interview, an invitation email by (M.B. and A.B.) was sent to the participants, in which the objectives of the study and related upstream documents were sent. At the beginning of the interviews, we explained the details of the interview and the reason for choosing the participant. |
| 8. Interviewer characteristics | What characteristics were reported about the inter viewer/facilitator? e.g. Bias, assumptions, reasons and interests in the research topic | Page 7 |

| **Domain 2: study design** |  |  |
| --- | --- | --- |
| *Theoretical framework* | | |
| 9. Methodological orientation and Theory | What methodological orientation was stated to underpin the study? e.g. grounded theory, discourse analysis, ethnography, phenomenology, content analysis | Page 7  Content analysis |
| *Participant selection* | | |
| 10. Sampling | How were participants selected? e.g. purposive, convenience, consecutive, snowball | Page 6  Based on the objectives of the study, we used purposive sampling to select individuals. we used snowball sampling. To maximize the data collected, we also interviewed as many participants as possible. |
| 11. Method of approach | How were participants approached? e.g. face-to-face, telephone, mail, email | Page 7  Due to the COVID-19 pandemic, we had to have the interviews on Skype. |
| 12. Sample size | How many participants were in the study? | Page 7  A total of 45 participants were interviewed. |
| 13. Non-participation | How many people refused to participate or dropped out? Reasons? | Page 7  Some people did not participate in the study due to being too busy, not having time for interviews, and lack of interest. |
| *Setting* | | |
| 14. Setting of data collection | Where was the data collected? e.g. home, clinic, workplace | Page 7  We interviewed the participants based on their preference at their workplace or home. |
| 15. Presence of non-participants | Was anyone else present besides the participants and researchers? | No. Page 7  M.B., A.B., and M.K.G. at the beginning of the interviews, we explained the details of the interview and the reason for choosing the participant. Due to the COVID-19 pandemic, we had to have the interviews on Skype. |
| 16. Description of sample | What are the important characteristics of the sample? e.g. demographic data, date | Page 7  Table 1 |
| *Data collection* | | |
| 17. Interview guide | Were questions, prompts, guides provided by the authors? Was it pilot tested? | Page 6  For pilot questions, and to meet the validity and reliability criteria, we sent out the texts of the interviews to five experts (see **Appendix 1**). They approved the questions and they were used in the interviews. |
| 18. Repeat interviews | Were repeat inter views carried out? If yes, how many? | Page 7  No repeat interviews carried out. |
| 19. Audio/visual recording | Did the research use audio or visual recording to collect the data? | Page 7  With the consent of the participants, we recorded interviews in Skype. |
| 20. Field notes | Were ﬁeld notes made during and/or after the interview or focus group? | Page 7  All interviews were recorded through field notes and participants' voices. |
| 21. Duration | What was the duration of the inter views or focus group? | Page 7  The interviews lasted between 45 and 75 minutes with an average duration of 55 minutes and continued until data saturation was reached. |
| 22. Data saturation | Was data saturation discussed? | Page 7  The interviews continued until data saturation was reached. |
| 23. Transcripts returned | Were transcripts returned to participants for comment and/or correction? | Page 7  All interview transcripts were sent to the participants to confirm their accuracy and to ensure credibility. |
| **Domain 3: analysis and ﬁndings** |  |  |
| *Data analysis* | | |
| 24. Number of data coders | How many data coders coded the data? | Page 8  The transcripts of interviews were scrutinized by four authors ( A.B., S.A, N.S, and S.M.) of the present study; then, the data were coded according to the four domains of SWOT (strengths, weaknesses, opportunities, and threats). |
| 25. Description of the coding tree | Did authors provide a description of the coding tree? | No explicit coding tree was used. |
| 26. Derivation of themes | Were themes identiﬁed in advance or derived from the data? | Page 8  Table 2  All the steps including collecting, analyzing, coding, and placing based on the four SWOT items. |
| 27. Software | What software, if applicable, was used to manage the data? | Page 8  Data processing was performed using the MAXQDA software. In the next step, we entered transcribed interviews into the software. |
| 28. Participant checking | Did participants provide feedback on the ﬁndings? | Page 7  All interview transcripts were sent to the participants to confirm their accuracy and to ensure credibility and their feedback was collected. |
| *Reporting* | | |
| 29. Quotations presented | Were participant quotations presented to illustrate the themes/ﬁndings? Was each quotation identiﬁed? e.g. participant number | Page 8  Table 3 to 6. |
| 30. Data and ﬁndings consistent | Was there consistency between the data presented and the ﬁndings? | Yes, there was consistency between data presented and findings, see pages 9-16 pages and table 2 to 6. |
| 31. Clarity of major themes | Were major themes clearly presented in the ﬁndings? | Page 8  Table 2 to 6. |
| 32. Clarity of minor themes | Is there a description of diverse cases or discussion of minor themes? | Page 8 , 9  Table 2 to 6. |
